# An Evaluation of the Knowledge and Utilization of the Partogragh in Primary, Secondary, and Tertiary Care Settings in Calabar, South-South Nigeria

**DOI:** 10.1155/2014/105853

**Published:** 2014-09-14

**Authors:** Ita B. Okokon, Afiong O. Oku, Thomas U. Agan, Udeme E. Asibong, Ekere J. Essien, Emmanuel Monjok

**Affiliations:** ^1^Department of Family Medicine, Faculty of Medicine and Dentistry, College of Medical Sciences, University of Calabar and University of Calabar Teaching Hospital, Calabar, Nigeria; ^2^Department of Community Medicine, Faculty of Medicine and Dentistry, College of Medical Sciences, University of Calabar and University of Calabar Teaching Hospital, Calabar, Nigeria; ^3^Department of Obstetrics and Gynecology, Faculty of Medicine and Dentistry, College of Medical Sciences, University of Calabar and University of Calabar Teaching Hospital, Calabar, Nigeria; ^4^Institute of Community Health, University of Houston, Texas Medical Center, Houston, TX, USA

## Abstract

The challenge to maternal well-being with associated maternal wastages especially in labor has remained unsurmountable across the three tiers of health care delivery in Nigeria. This study aimed to determine and compare the factors that influence utilization of the partograph in primary, secondary, and tertiary health care delivery levels in Calabar, Nigeria. This was a descriptive study, using a self-administered semistructured questionnaire on 290 consenting nonphysician obstetric care workers, purposively recruited. The mean age of the respondents was 40.25 ± 8.68 with a preponderance of females (92.4%). Knowledge of the partograph and previous partograph training had statistically significant relationship with its utilization among respondents from the tertiary and general hospitals. The level of knowledge was higher among workers in the general hospital than those working in the university teaching hospital. Nurses/midwives in the three levels of care were significantly more knowledgeable in partograph use than other nonphysician obstetric care workers. Lack of detailed knowledge of the partograph, its nonavailability and poor staff strength in the study centers were factors militating against its ease of utilization. The authors recommend periodic in-service training and provision of partograph in labor rooms in all maternity wards in our environment.

## 1. Introduction

Prolonged obstructed labor is one of the major direct causes of maternal mortality in Nigeria, preceded only by obstetric hemorrhage, sepsis (abortion), and toxemia of pregnancy, in that order [1, 2]. Indirect causes of maternal deaths are conditions which complicate preexisting maternal illnesses or disease conditions. They emerge in the course of the pregnancy, like malaria, viral hepatitis, and human immunodeficiency virus infection/acquired immunodeficiency syndrome (HIV/AIDS). It has been reported that about 1-2% of obstructed labor are mechanically caused in the second stage of labor [3]. In 2005, the World Health Organization (WHO) estimated maternal mortality rate (MMR) for Nigeria to be 1,100 deaths per 100,000 live births [4]. However, a more recent facility estimate (2010) of MMR for Calabar metropolis stood at 1,500 per 100,000 live births [5]. The latter figure is astronomically wide from the WHO recent global MMR estimate (2011) of 840 deaths per 100,000 live births [6], with the obvious implications that coping mechanisms for obstetric patients in labor, possibly in terms of availability of trained man-power, befitting healthcare facilities, government funding to maternity services, and other indirect contending issues like economic circumstances of the people and means of transportation, have all depreciated remarkably in Calabar.

Maternal mortality occasioned by obstructed labor is preventable and there are convincing reports that the acquisition of the knowledge and utilization of the partograph, would result in an impressive fall in its incidence, which contributes about 8–10% of maternal deaths [7–9].

The partogram was introduced through the pioneering work of Philpott and Castle in 1972 [9], used convincingly by Studd in 1973 [10], and later modified and adapted for global usage in all settings by the WHO in 1988 [9], hence its conceived applicability in this study. It is a graphic representation of the events of labor, depicting maternal and fetal circumstances, plotted against time in hours [7–9]. Correct utilization of the partograph will stem prolonged and/or obstructed labor in all settings, thus reducing significantly the sequelae of maternal and fetal wastages [3, 9].

Maternal mortality has been extensively studied in Calabar with very poor maternal mortality rates in both situations [5, 11]. There is as yet no study that compares knowledge and utilization of the partograph among the different user groups in this environment, to underscore the proper applicability of this basic labor monitoring instrument or the lack of it. The current study was therefore carried out to determine and compare the factors that influence the utilization of the partograph—a simple labor monitoring tool—among nonphysician obstetric care workers in the primary, secondary, and tertiary healthcare delivery levels in Calabar, Nigeria.

The range of obstetric care workers (OCWs) in tertiary and secondary healthcare delivery levels in the urban settings in Nigeria is wide. It includes obstetricians, family physicians, general duty doctors, nurses and midwives, auxiliary nurses, community health officers, and community health extension workers (CHEWS). On the contrary, at the primary care level, the physician compliment of this list is often lacking while the inclusion of nurse aids, junior community health extension workers (JCHEWS), and traditional birth attendants (TBAs) becomes evident.

Studying the activities of OCWs with regard to their knowledge and utilization of the partograph at the primary care level side by side with the secondary and tertiary levels in Calabar, therefore, would readily unveil some of the reasons for maternal wastages which have continued to be an unsurmountable problem in our environment till date.

The results of this study should provide scientific data that would enable the formulation of policies aimed at improving MMR in our environment, through continuing medical and nursing professional development on obstetric care giving, in order to meet the United Nations Millennium goal 5, slated for 2015. Furthermore, this study opens a lacunar of challenge for the family physician in that he is the medical expert whose services can be seen in the three tiers of healthcare delivery in the obstetric care environment in our setting: as a resident doctor in the tertiary healthcare system, as the officer-in-charge of the system in some secondary healthcare delivery facilities, and as the one to receive referrals from primary care centers in such secondary settings in obstetric care giving.

## 2. Materials and Methods

### 2.1. Study Setting

This study was conducted in the Department of Family Medicine, University of Calabar Teaching Hospital (UCTH), Calabar, Nigeria. It involved the Maternity Unit of the Obstetrics and Gynecology Department of the UCTH as a tertiary healthcare center; the General Hospital (GH), Calabar, as a secondary healthcare center; and three primary healthcare (PHC) centers, in Calabar.

The UCTH is the only tertiary healthcare delivery center in Calabar with one hundred and ten obstetric beds and over 2000 deliveries are taken here annually. The General Hospital in Calabar is the only government owned secondary healthcare facility in the metropolis and the maternity unit has twenty-four of the total of its one hundred bed capacity. It takes an average of 800 deliveries annually. The three PHC centers used in this study, which were determined by random selection, are among the twelve in the metropolis. Their bed capacities are five, seven, and eight, and they take an average of eighty-four, one hundred and eight, and one hundred and twenty deliveries annually, respectively.

Calabar is the capital of the Cross River State of Nigeria. It lies along latitude 4°58′ north of the equator and longitude 8°20′ east of the Greenwich Meridian [12], within the South-South geopolitical zone of the country. It has a population of 372,848 by the Nigerian national population and housing census of 2006 [13].

### 2.2. Study Design

This study was a descriptive study using self-administered semistructured questionnaire designed to determine the knowledge and utilization of the partograph in the three levels of healthcare in Calabar, Nigeria.

### 2.3. Study Population

Two hundred and ninety (290) consenting nonphysician obstetric care workers (OCWs) were recruited for the study in all three tiers of the healthcare delivery system: primary, secondary, and tertiary in the Calabar metropolis.

All nonphysician obstetric care workers in the Department of Obstetrics and Gynecology of the University of Calabar Teaching Hospital who consented were purposively recruited to participate in the study. Informed consent was obtained from the 137 respondents from the UCTH before they were served with the questionnaire. Of the 137 that received the questionnaire, 132 responses were realized. The same procedure was followed at the General Hospital, Calabar, and out of 133 consenting respondents who were served with the questionnaire, 130 responded. Of the 12 PHCs in Calabar metropolis, three were randomly selected and 30 consenting nonphysician OCWs were served with the questionnaire, out of which 28 responded. The return rate of questionnaires from the three levels of care was 96.7% (290).

### 2.4. Analysis of Data

Data obtained from this study were analyzed using SPSS for windows version 19.0. Descriptive statistics (frequencies, proportions, means, percentages, tables, and standard deviation) were used to summarize variables, while inferential statistics (chi square test) was used to test the significance of association between categorical variables with the level of significance set at 5%. The test-retest reliability of the questionnaire had been carried out in a previous study [14]. The evaluation of the knowledge score also used that derived from the same study [14]. Consequently, a score of >12 was rated as good knowledge and a score <12 was rated as poor knowledge of the partograph (Table 9).

### 2.5. Ethical Issues

Ethical issues on this study were addressed by the University of Calabar Teaching Hospital Health Research Ethics Committee and the Health Research Ethics Committee of the Cross River State Ministry of Health, Calabar.

## 3. Results

Two hundred and ninety (290) consenting nonphysician obstetric care workers (OCWs) participated in this study. Forty-five point five percent (45.5%) of this number was drawn from the University Teaching Hospital (tertiary center), 44.8% from the General Hospital (secondary center), and 9.7% from three primary healthcare centers in Calabar metropolis.

Table 1 shows the demographic profile of the subjects. The age range of the subjects spanned 24 to 58 years. There was a female preponderance at a ratio of 12 : 1 with 77.5% of the total female subjects being nurse/midwife. Three of the subjects were less than one year in the service while ten were getting near their retirement duration in service (35 years).

Respondents' knowledge of the definition of the partograph is shown in Table 2. About two-thirds of the respondents in the three levels of care were unable to identify the correct definition of the partograph as a simple graphic recording of labor and salient conditions of the mother and fetus against time in hours.

In Table 3, which assessed the use of the partograph as a preventive tool, the results show that 62.4% of the participants indicated that it can reduce maternal morbidity, 73.8% indicated that it can reduce maternal mortality, 53.4% indicated that it can reduce child morbidity and 69.3% indicated it can reduce newborn mortality. However, a larger percentage of the respondents, majority of them, nurses/midwives who did not know about these preventive functions, were working at the tertiary level of care.

A detailed assessment of the graphic representation of normal labor on the partograph was also done (Table 4). The results show very poor knowledge of the graphic representation. Many of them did not know the correct graphic patterns. For example, only 29% knew that the graph should fall to the left of the alert line and 49% did not know.

The category of assessment made with the partograph was also studied (Table 5). Most of the respondents indicated that the partograph can be used in detecting prolonged labor (71.0%), poor progress of labor (71.0%), insufficient uterine contractions (62.1%), satisfactory progress of labor (62.8%), suspected fetal distress (60.7%), need for labor augmentation (60.3%), abnormal fetal heart rate (58.6%), need for Caesarean delivery (57.2%), obstructed labor (56.6%), and dehydration in the mother (41.0%). The knowledge base of the respondents (mainly nurses and midwives) was consistently greater among the workers in the General Hospital (a secondary health facility) than the University Teaching Hospital, a tertiary health facility.

Factors militating against the use of the partograph in labor monitoring at all levels of healthcare (Table 6) were as follows: little or no knowledge (79.0%), nonavailability of the partograph (58.6%), shortage of staff (46.9%), and time consuming (24.1%).

The relationship between knowledge, years of experience with the utilization of the partograph within the practice level, and partograph availability is represented in Table 7. Knowledge of the partograph had a significant relationship with its utilization among workers in UCTH (*χ* ^2^ = 38.96, *p* ≤ 0.0001) and General Hospital (*χ* ^2^ = 12.05, *p* ≤ 0.0001). Partograph availability also had a statistically significant relationship with its utilization in the three levels of health facility, UCTH (*χ* ^2^ = 52.5, *p* ≤ 0.0001), General Hospital (*χ* ^2^ = 56.5, *p* ≤ 0.0001), and PHC facilities (*p* ≤ 0.0001). The relationship between years of experience and utilization was not statistically significant.

Table 8 shows that nurse/midwife working at all levels were more knowledgeable on the use of the partograph compared to other OCWs: UCTH (*χ* ^2^ = 26.5, *p* ≤ 0.0001), General Hospital (*χ* ^2^ = 7.44, *p* ≤ 0.006), and PHC (Fisher's exact test; *p* = 0.004). Also, there was a statistically significant relationship between previous partograph training among UCTH (*χ* ^2^ = .4.31, *p* ≤ 0.04) and GH (*χ* ^2^ = 9.43, *p* ≤ 0.002) obstetric care workers. The PHC workers' previous training (Fisher's exact test: *p* = 0.43) did not have any significant relationship with their knowledge. Age and years of experience in partograph utilization showed no statistically significant associations.

## 4. Discussion

In this study, knowledge of the partograph and its availability had a significant relationship with its utilization. This finding is similar to studies conducted in Ogun State, in South West Nigeria [15], Bayelsa in South-South Nigeria [14], and Enugu in South East Nigeria [16]. Also, previous training on the partograph in school and being a trained nurse/midwife show significant association with a better overall knowledge of the partograph. This has been identified in other studies where nurses at the tertiary levels had better knowledge due to periodic seminars and in-service training compared to their colleagues at the private health facilities and PHC centers [14–17].

This study identified the following as factors militating against the use of the partograph: little or no knowledge on the partograph (79%), nonavailability of the instrument in labor wards (58.6%), and shortage of staff (46.9%). Only 24.1% indicated that it was time consuming. Interestingly, 75.9% indicated that it was not time consuming, which seems a contradiction because with staff shortages, an additional task of using the partograph would normally be seen as time consuming especially if the benefits of the instrument are not well appreciated. This finding is significant and calls for hospital and Ministry of Health officials to make sure the instrument, a cost effective tool in labor monitoring, is made available in all maternity centers.

This study used nonphysician obstetric care workers from the three levels of healthcare in Calabar metropolis. The reason is because these categories of workers, especially nurses and midwives form the bulk of skilled birth attendants in Nigeria and their knowledge and utilization of this simple preventive tool will go a long way in reducing maternal and neonatal morbidity and mortality. Nigeria is one of the six countries in the world contributing about 10% to global maternal mortality statistics and also one of the countries that appears not to be on the road map of achieving the MDG 5 in 2015 [18].

Many of the respondents (70.3%) were not able to correctly define the partograph even when the questions made available to them were quite simple. Also, the detailed knowledge of the component parts of the partograph with regard to normal labor tracing, its relationship to the alert and action lines, and number of uterine contractions and duration of strong contractions was very poor indeed. Several studies in Nigeria also indicate the general overall poor knowledge of the partograph [14–17]. Interestingly, the workers at the General Hospital (the secondary healthcare facility) had a better overall knowledge than those at the University Teaching Hospital, a tertiary health facility. This finding is not in agreement with some studies in South West Nigeria [15, 17] where the knowledge was higher among tertiary health workers. However, a study in Ethiopia did show that workers in the health centers had good knowledge and better utilization than workers in the hospital [19]. Since the partograph has been recommended by WHO as a preventive tool in labor monitoring in all public institutions [9, 20], especially in low resources countries, it is important that these health workers be adequately knowledgeable on which aspect of prevention the partograph is most useful. In this regard, this study also showed that workers at the secondary health facility had overall higher knowledge on the benefit of this instrument with respect to assessment for prolonged labor, obstructed labor, poor progress of labor, insufficient uterine contractions, suspecting fetal distress, abnormal fetal heart rate, the need for labor augmentation, and Caesarean deliveries than those working at the University Teaching Hospital. The likely explanation for this is that health workers at this level use the instrument more regularly to identify abnormal labor patterns early so they could arrange for timely referral to the doctor since nurses and midwives are the primary birth attendants in all maternities at the primary and secondary health facilities in Nigeria. This finding was also corroborated by Yisma et al. in Ethiopia [19].

## 5. Limitations of the Study

The smallness of the sample size, especially from the PHC, would make the estimates and the dependent and independent variables unstable and therefore undetected. This could make the study appear as lacking in limitation. However, a larger size from the PHC in the entire state (geopolitical zone/local government areas) will be a good study among PHC workers; many of them are nurse aids, CHEWs, JCHEWs, and ward orderlies, who form the bulk of the unskilled birth attendants in Nigeria. Secondly, the study does not include obstetric care workers in private hospitals, so, the findings cannot be generalized for Calabar metropolis. Thirdly, distortions can occur with the use of self-administered questionnaires; the social desirability bias where the respondents will tend to give answers they feel is socially desirable to the researchers, in case this information will get to the authorities at the Health Ministry.

## 6. Conclusion and Recommendations

This study implicates lack of detailed knowledge of the partograph, nonavailability, and poor staff strength as factors militating against optimal utilization of this very important preventive labor monitoring tool in Calabar metropolis. The importance of training and experience is highlighted in this study since an association between good knowledge and utilization was established. Working in the secondary healthcare level and the PHC was related to having overall higher knowledge of the partograph in this study. This means that OCWs at these two levels utilized the partograph more than their counterparts in the teaching hospital, possibly because they needed it to guide them in early referral which is not necessarily a problem in the teaching hospital.

The findings of this study underline the fact that the knowledge and inclination to use this instrument should be reinforced through periodic regular professional education by way of unit presentations, seminars, and workshops. The partograph charts should be made available by the hospital management for use at all times in the labor rooms in line with WHO recommendation and Safe Motherhood programs.

The authors recommend periodic in-service training for all OCWs, the provision of partographs in labor rooms in all maternities in the study environment, and regular supervision and mandatory health facility policies from the hospital administration regarding the use of the partograph (tertiary healthcare), State Ministry of Health (secondary healthcare), and local government health departments (PHC clinics) for the safety of mothers in Calabar metropolis as also expected for all other parts of Nigeria.

## Figures and Tables

**Table 1:** Demographic profile of the subjects.

	UCTH (3^0^) *N* = 132 Freq. (%)	GHC (2^0^) *N* = 130 Freq. (%)	PHCs *N* = 28 Freq. (%)	Total *N* = 290
Age				
≤29	29 (22.0)	9 (6.9)	2 (7.1)	40 (13.8)
30–39	44 (33.3)	28 (21.5)	11 (39.3)	88 (28.6)
40–49	40 (30.3)	71 (54.7)	11 (39.3)	122 (42.1)
>50	19 (14.4)	22 (16.9)	4 (14.3)	45 (15.5)
Total	**132** (**100**)	**130** (**100**)	**28** (**100**)	**290** (**100**)
Mean (SD)	37.84 ± 9.38	42.45 ± 7.53	41.39 ± 7.40	40.25 ± 8.68
Sex				
Male	17 (12.9)	3 (2.3)	2 (7.1)	22 (7.6)
Female	115 (87.1)	127 (97.7)	26 (92.9)	268 (92.4)
Total	**132** (**100**)	**130** (**100**)	**28** (**100**)	**290** (**100**)
Length of time in practice				
1–5	31 (23.5)	12 (9.2)	2 (7.1)	45 (15.6)
6–10	30 (22.7)	8 (6.2)	2 (7.1)	40 (13.8)
11–15	23 (17.4)	18 (13.8)	7 (25.0)	48 (16.6)
16–20	23 (17.4)	26 (20.0)	7 (25.0)	56 (19.3)
>20	25 (18.9)	66 (50.8)	10 (33.7)	101 (34.8)
Total	**132** (**100**)	**130** (**100**)	**28** (**100**)	**290** (**100**)
Health worker cadre				
Nurse/midwife	109 (82.6)	102 (78.5)	14 (50.0)	225 (77.5)
CHEW	14 (10.6)	8 (6.2)	6 (21.4)	28 (9.7)
Nurse aid	9 (6.8)	8 (6.2)	3 (10.7)	20 (6.9)
Others∗	0 (0)	12 (9.2)	5 (17.9)	17 (5.9)
Total	**132** (**100**)	**130** (**100**)	**28** (**100**)	**290** (**100**)

*Others included JCHEWS and community aids.

**Table 2:** Respondents knowledge of partograph.

Variables	UCTH (3^0^) *N* = 132 Freq. (%)	GHC (2^0^) *N* = 130 Freq. (%)	PHCs (1^0^) *N* = 28 Freq. (%)	Total *N* = 290
A chart developed by midwives in developed countries to monitor labor				
Yes	22 (16.7)	5 (3.8)	0 (0)	27 (9.3)
No	110 (83.3)	125 (96.2)	28 (100)	263 (90.7)
Total	**132** (**100**)	**130** (**100**)	**28** (**100**)	**290** (**100**)
A complex tool with pictorial overview of labor for use by midwives				
Yes	14 (10.6)	14 (10.8)	1 (3.6)	29 (10.0)
No	118 (89.4)	116 (89.2)	27 (96.4)	261 (90.0)
Total	**132** (**100**)	**130** (**100**)	**28** (**100**)	**290** (**100**)
A chart for monitoring labor by doctors				
Yes	24 (18.2)	7 (5.4)	6 (21.4)	37 (12.8)
No	108 (81.8)	123 (94.6)	22 (78.6)	253 (87.2)
Total	**132** (**100**)	**130** (**100**)	**28** (**100**)	**290** (**100**)
A simple graphic recording of labor and salient conditions of the mother and fetus against time in hours				
Yes	51 (38.6)	28 (21.5)	7 (25.0)	86 (29.7)
No	81 (61.4)	102 (78.5)	21 (75.0)	204 (70.3)
Total	**132** (**100**)	**130** (**100**)	**28** (**100**)	**290** (**100**)

**Table 3:** Knowledge of partograph as preventive tool.

Knowledge of partograph	UCTH (3^0^) *N* = 132 Freq. (%)	GHC (2^0^) *N* = 130 Freq. (%)	PHCs (1^0^) *N* = 28 Freq. (%)	Total *N* = 290
Reduces maternal morbidity				
Yes	76 (57.6)	86 (66.2)	19 (67.9)	181 (62.4)
No	21 (15.9)	43 (33.1)	9 (32.1)	73 (25.2)
Do not know	35 (26.5)	1 (0.8)	0 (0)	36 (12.4)
Reduces maternal mortality				
Yes	94 (71.2)	97 (74.6)	23 (82.1)	214 (73.8)
No	23 (17.4)	32 (24.6)	5 (17.9)	60 (20.7)
Do not know	15 (11.4)	1 (0.8)	0 (0)	16 (5.5)
Reduces child morbidity				
Yes	74 (56.1)	66 (46.2)	15 (53.6)	155 (53.4)
No	31 (23.5)	60 (50.8)	11 (39.3)	102 (35.2)
Do not know	27 (20.5)	4 (3.1)	2 (7.1)	33 (11.4)
Reduces newborn mortality				
Yes	86 (65.2)	95 (73.1)	20 (71.4)	201 (69.3)
No	11 (8.3)	34 (26.2)	7 (25.0)	52 (17.9)
Do not know	35 (26.5)	1 (0.8)	1 (3.6)	37 (12.8)

**Table 4:** Knowledge of labor: detailed graphic representation.

Knowledge of labor	UCTH (3^0^) *N* = 132 Freq. (%)	GHC (2^0^) *N* = 130 Freq. (%)	PHCs (1^0^) *N* = 28 Freq. (%)	Total *N* = 290		
The graph on the partograph to fall to the left of the alert line						
No	36 (27.3)	13 (10)	15 (53.6)	64 (22.1)		
yes	49 (37.1)	31 (23.8)	4 (14.3)	84 (29.0)	43.4	<0.0001
Do not know	47 (35.6)	86 (66.2)	9 (32.1)	142 (49.0)		
The graph on the partograph to fall on the alert line						
Yes	34 (25.8)	23 (17.7)	5 (17.9)	62 (21.4)		
No	36 (27.3)	11 (8.5)	16 (57.1)	63 (21.7)	44.6	<0.0001
Do not know	62 (47.0)	96 (73.8)	7 (25.0)	165 (56.9)		
The graph on the partograph to fall to the right of the alert line						
Yes	41 (31.1)	33 (25.4)	4 (14.3)	78 (26.9)		
No	41 (31.1)	7 (5.4)	17 (60.7)	65 (22.4)	58.9	<0.0001
Do not know	50 (37.9)	90 (69.2)	7 (25.0)	147 (50.7)		
During labor 3 contractions being observed every 10 minutes						
Yes	74 (56.1)	58 (44.6)	9 (32.1)	46 (15.9)		
No	14 (10.6)	15 (11.5)	17 (60.7)	141 (48.6)	51.9	<0.0001
Do not know	44 (33.3)	57 (43.8)	2 (7.1)	103 (35.5)		
Minimum duration of a strong contraction being 40 seconds						
Yes	53 (40.2)	51 (39.2)	7 (25.0)	111 (38.3)		
No	25 (18.9)	12 (9.2)	4 (14.3)	41 (14.1)		
Do not know	54 (40.9)	67 (51.5)	17 (60.7)	138 (47.6)	8.49	0.075
10 minutes being required to adequately assess contractions						
Yes	66 (12.1)	45 (34.6)	12 (42.9)	123 (42.4)		
No	16 (12.1)	19 (14.6)	2 (7.1)	37 (12.8)		
Do not know	50 (37.9)	66 (50.8)	14 (50.1)	130 (44.8)	7.35	0.11

**Table 5:** Assessment made with the partograph.

Assessment made with the partograph	UCTH (3^0^) *N* = 132 Freq. (%)	GHC (2^0^) *N* = 130 Freq. (%)	PHC (1^0^) *N* = 28 Freq. (%)	Total *N* = 290	*χ* ^2^	*p* value
Prolonged labor						
Yes	92 (69.7)	95 (73.1)	19 (67.9)	206 (71.0)		
No	40 (30.3)	35 (26.9)	9 (32.1)	84 (29.0)	0.52	0.77
Obstructed labor						
Yes	70 (53.0)	84 (64.6)	10 (35.7)	164 (56.6)		
No	62 (47.0)	42 (35.4)	18 (64.3)	126 (43.4)	9.05	**0.011**
Poor progress of labor						
Yes	83 (62.9)	105 (80.8)	18 (64.3)	206 (71.0)		
No	49 (37.1)	25 (19.2)	10 (35.7)	84 (29.0)	10.9	**0.004**
Inefficient uterine contraction						
Yes	75 (56.8)	89 (68.5)	16 (57.1)	180 (62.1)		
No	57 (43.2)	41 (31.5)	12 (42.9)	119 (37.9)	4.09	0.13
Suspected fetal distress						
Yes	70 (53.0)	89 (68.5)	17 (60.7)	176 (60.7)		
No	62 (47.0)	41 (31.5)	11 (39.3)	114 (39.3)	6.53	**0.038**
Abnormal FHR						
Yes	70 (53.0)	87 (66.9)	13 (46.4)	170 (58.6)		
No	62 (47.0)	43 (33.1)	15 (53.6)	120 (41.4)	7.11	**0.03**
Satisfactory progress of labor						
Yes	78 (59.1)	89 (68.5)	15 (53.6)	182 (62.8)		
No	54 (40.9)	41 (31.5)	13 (46.4)	108 (37.2)	3.58	0.17
Need for labor augmentation						
Yes	79 (59.8)	85 (65.4)	11 (39.3)	175 (60.3)		
No	53 (40.2)	45 (34.6)	17 (60.7)	115 (39.7)	6.58	**0.037**
Need for Caesarean delivery						
Yes	74 (56.1)	83 (63.8)	9 (32.1)	166 (57.2)		
No	58 (43.9)	47 (36.2)	19 (67.9)	124 (42.8)	9.60	**0.008**
Dehydration in mother						
Yes	45 (34.1)	68 (52.3)	16 (57.1)	119 (41.0)		
No	87 (65.9)	62 (47.7)	12 (42.9)	171 (59.0)	5.05	0.08

**Table 6:** Factors militating against use of the partograph, at all levels.

Factors	UCTH (3^0^) *N* = 132 Freq. (%)	GHC (2^0^) *N* = 130 Freq. (%)	PHC (1^0^) *N* = 28 Freq. (%)	Total *N* = 290	*χ* ^2^	*p* value
Little or no knowledge						
Yes	97 (73.5)	111 (85.4)	21 (75.0)	229 (79.0)		
No	35 (26.5)	19 (14.6)	7 (25.0)	61 (21.0)	5.88	0.05
Nonavailability						
Yes	61 (46.2)	91 (70.0)	18 (64.3)	170 (58.6)		
No	71 (53.8)	39 (30.0)	10 (35.7)	120 (41.4)	15.7	**<0.0001**
Shortage of staff						
Yes	46 (34.8)	80 (61.5)	10 (35.7)	136 (46.9)		
No	86 (65.2)	50 (38.5)	10 (35.7)	154 (53.1)	20.3	**<0.0001**
Time consuming						
Yes	26 (19.7)	39 (30.0)	5 (17.9)	70 (24.1)		
No	106 (80.3)	91 (70.0)	23 (82.1)	220 (75.9)	4.46	0.11

**Table 7:** Relationship between knowledge, years of experience in utilization of partograph, and availability within practice levels.

	Utilization of partograph					
	UCTH *n* = 132		GHC = 130		PHC *n* = 28	
	Yes (%)	No (%)	Yes (%)	No (%)	Yes (%)	No (%)
Knowledge						
Good	68 (88.3)	9 (11.7)	55 (59.8)	37 (40.2)	8 (44.4)	10 (55.6)
Poor	20 (36.4)	35 (63.6)	10 (26.3)	28 (73.7)	5 (50.0)	5 (50.0)
Total	**88** (**66.7**)	**44** (**33.3**)	**65** (**50.0**)	**65** (**50.0**)	**13** (**46.4**)	**15** (**53.6**)
	*p* ≤ 0.0001 *χ* ^2^ = 38.96		*p* ≤ 0.0001 *χ* ^2^ = 12.05		*p* = 0.778 *χ* ^2^ = 0.08	
Years of experience						
≤**15**	45 (73.8)	16 (26.2)	10 (50.0)	10 (50.0)	0 (0)	41 (100)
>15	43 (60.6)	28 (39.4)	55 (50.0)	55 (50.0)	13 (54.2)	11 (45.8)
Total	**88** (**66.7**)	**44** (**33.3**)	**65** (**50.0**)	**65** (**50.0**)	**13** (**46.4**)	15 (53.8)
	*p* = 0.11 *χ* ^2^ = 2.58		*p* = 1.00 *χ* ^2^ = 0.00		*p* = 0.10	
Partograph availability						
No	15 (28.8)	37 (71.2)	47 (90.4)	5 (9.6)	11 (91.7)	1 (8.3)
Yes	73 (91.3)	7 (8.8)	18 (23.1)	60 (76.9)	2 (12.5)	14 (87.5)
Total	88 (66.7)	44 (33.3)	65 (50.0)	65 (50.0)	13 (46.4)	15 (53.6)
	*χ* ^2^ = 52.5 *p* ≤ 0.0001		*χ* ^2^ = 56.5 *p* ≤ 0.0001		*p* ≤ 0.0001	

**Table 8:** Relationship between demographics and knowledge within practice levels.

Variable	Overall knowledge					
	UCTH (3^0^) *n* = 132		GHC (2^0^) = 130		PHC (1^0^) *n* = 28	
	Good (%)	Poor (%)	Good (%)	Poor (%)	Good (%)	Poor (%)
Sex∗∗						
Female	72 (62.6)	43 (37.4)	90 (70.9)	37 (29.1)	17 (65.4)	9 (50.0)
Male	5 (29.4)	12 (70.6)	2 (66.7)	1 (33.3)	1 (50.0)	1 (50.0)
Total	**77** (**58.3**)	**55** (**41.7**)	**92** (**70.8**)	**38** (**29.2**)	**18** (**64.3**)	**10** (**35.7**)
	*p* = 0.01 *χ* ^2^ = 6.72		*p* = 1.00		*p* = 1.00	
Cadre∗∗						
**Nurse**/**midwife**	73 (69.5)	32 (30.5)	78 (76.5)	24 (23.5)	13 (92.9)	1 (7.1)
Other HCW	4 (14.8)	23 (85.2)	14 (50.0)	14 (50.0)	5 (35.7)	9 (64.3)
	77 (58.3)	55 (41.7)	92 (70.8)	38 (29.2)	18 (64.3)	10 (35.7)
	*p* ≤ 0.0001 *χ* ^2^ = 26.5		*p* = 7.44 *χ* ^2^ = 0.006		*p* = 0.004	
Age∗∗						
≤35	40 (70.2)	17 (29.8)	11 (55.0)	9 (45.0)	2 (40.0)	3 (60.0)
>35	37 (49.3)	38 (50.7)	81 (73.6)	29 (26.4)	16 (69.6)	7 (30.4)
	77 (58.3)	55 (41.7)	92 (70.8)	38 (29.2)	18 (64.3)	10 (35.7)
	*χ* ^2^ = 5.79 *p* = 0.016		*χ* ^2^ = 2.84 *p* = 0.09		*p* = 0.32	
Previous training∗∗						
No	28 (48.3)	30 (51.7)	29 (58.3)	23 (44.2)	97 (75.0)	3 (25.0)
Yes	49 (66.2)	25 (33.8)	63 (80.8)	15 (19.2)	9 (56.3)	7 (43.8)
Total	**77** (**58.3**)	**55** (**41.7**)	**92** (**70.8**)	**38** (**29.2**)	**18** (**64.3**)	**10** (**35.7**)
	*χ* ^2^ = 4.31 *p* = 0.04		*χ* ^2^ = 9.43 *p* = 0.002		*p* = 0.43	
Years of experience∗∗						
5 years and less	21 (67.7)	10 (32.3)	8 (66.7)	4 (33.3)	2 (100.0)	0 (0)
Over 5 years	56 (55.4)	45 (44.6)	84 (71.2)	34 (28.8)	16 (61.5)	10 (38.5)
Total	77 (58.3)	55 (41.7)	92 (70.8)	38 (29.2)	18 (64.3)	10 (35.7)
	*χ* ^2^ = 1.48 *p* = 0.22		*p* = 0.75		*p* = 0.52	

**Fisher's exact test used for PHC.

**Table 9:** Criteria for the partograph knowledge score.

	Knowledge assessment	Scoring
(1)	Correct definition of a partograph	4
(2)	Benefits derived from use of partograph	
(i)	Reduce maternal deaths	1
(ii)	Reduce maternal morbidity	1
(iii)	Reduce deaths in newborn	1
(iv)	Reduce child morbidity	1
(v)	Increase efficiency in labor	1
(vi)	Mandatory for improved quality of care	1
(3)	Correct mention of the parts of a partograph	
(i)	Fetal well-being	1
(ii)	Progress of labor	1
(iii)	Maternal well-being	1
(4)	Function of action line	3
(5)	Correct assessment of normal progress of labor	3
(6)	Components of labor assessment	
(i)	Number of contractions	1
(ii)	Duration of contraction	1
(iii)	Duration of labor	1
(iv)	Time required to assess adequacy of contractions	1
(v)	Progress of labor	1

	Total	24

Q 1, 4, and 5 were true/false options. A score of >12 was classified as having good knowledge while a score of <12 as poor knowledge.
